# Severity of chronic Lyme disease compared to other chronic conditions: a quality of life survey

**DOI:** 10.7717/peerj.322

**Published:** 2014-03-27

**Authors:** Lorraine Johnson, Spencer Wilcox, Jennifer Mankoff, Raphael B. Stricker

**Affiliations:** 1 LymeDisease.org, Chico, CA, USA; 2 Human-Computer Interaction Institute, Carnegie Mellon University, Pittsburgh, PA, USA; 3 International Lyme & Associated Diseases Society, Bethesda, MD, USA

**Keywords:** Lyme disease, HRQoL, *Borrelia burgdorferi*, Survey

## Abstract

**Overview**. The Centers for Disease Control and Prevention (CDC) health-related quality of life (HRQoL) indicators are widely used in the general population to determine the burden of disease, identify health needs, and direct public health policy. These indicators also allow the burden of illness to be compared across different diseases. Although Lyme disease has recently been acknowledged as a major health threat in the USA with more than 300,000 new cases per year, no comprehensive assessment of the health burden of this tickborne disease is available. This study assesses the HRQoL of patients with chronic Lyme disease (CLD) and compares the severity of CLD to other chronic conditions.

**Methods**. Of 5,357 subjects who responded to an online survey, 3,090 were selected for the study. Respondents were characterized as having CLD if they were clinically diagnosed with Lyme disease and had persisting symptoms lasting more than 6 months following antibiotic treatment. HRQoL of CLD patients was assessed using the CDC 9-item metric. The HRQoL analysis for CLD was compared to published analyses for the general population and other chronic illnesses using standard statistical methods.

**Results**. Compared to the general population and patients with other chronic diseases reviewed here, patients with CLD reported significantly lower health quality status, more bad mental and physical health days, a significant symptom disease burden, and greater activity limitations. They also reported impairment in their ability to work, increased utilization of healthcare services, and greater out of pocket medical costs.

**Conclusions**. CLD patients have significantly impaired HRQoL and greater healthcare utilization compared to the general population and patients with other chronic diseases. The heavy burden of illness associated with CLD highlights the need for earlier diagnosis and innovative treatment approaches that may reduce the burden of illness and concomitant costs posed by this illness.

## Introduction

Lyme disease is the most common vector-borne disease in the United States. It is caused by the spirochete *Borrelia burgdorferi* and transmitted via tick bite. In its early, or acute, form, the disease may cause a hallmark erythema migrans (EM) rash and/or flu-like symptoms such as fever, malaise, fatigue, and generalized achiness (Aucott et al., 2009). Unfortunately, many patients are not diagnosed early because the carrier tick may be as small as a poppy seed, its bite is painless, and the hallmark EM rash does not occur in a significant percentage of patients (Aucott et al., 2009).

The CDC estimates that roughly 300,000 people (approximately 1% of the U.S. population) are diagnosed with Lyme disease each year (Centers for Disease Control and Prevention, 2013b). This figure is 1½ times higher than the number of women diagnosed with breast cancer each year in the USA (approximately 200,000), (Centers for Disease Control and Prevention, 2013a) and 6 times higher than the number diagnosed with HIV/AIDS each year in the USA (50,000) (Centers for Disease Control and Prevention, 2013c). A proportion of patients with Lyme disease develop debilitating symptoms that persist in the absence of initial treatment or following short-course antibiotic therapy. This condition is commonly referred to as post-treatment Lyme disease (PTLD) or chronic Lyme disease (CLD). It is estimated that as many as 36% of those diagnosed and treated early for Lyme disease remain ill after treatment (Aucott et al., 2013).

Although the CDC is tracking the *spread* of Lyme disease, very little data is available about its *impact* on patient quality of life, healthcare service needs and work capability. The CDC has developed a broad health-related quality of life (HRQoL) metric that is included in numerous government population surveys and sets goals for Healthy People 2020 (US Department of Health and Human Services, 2011). The HRQoL metric is a 9-item survey (4-item Healthy Days Core Module and 5-item Healthy Days Symptoms Module) that is used to assess health in the general population, determine the symptom burden of chronic diseases, identify health disparities and unmet health needs, evaluate progress on achieving goals, and inform public health policy (Moriarty, Zack & Kobau, 2003). In addition, it permits the burdens of different diseases to be compared despite the widely varying time course, symptom patterns, functional impairment, and clinical severity associated with these diseases (Cook & Harman, 2008; Institute of Medicine, 2012). The patient-centered HRQoL indicators are considered to be robust predictors of subsequent health outcomes and health system utilization (DeSalvo et al., 2005).

The purpose of this study is to document the severity of CLD compared to other chronic conditions using the CDC HRQoL metric. In addition, we discuss HRQoL indicators in the context of physical and mental health, healthcare service utilization and work productivity. To our knowledge, this is the first study that examines these HRQoL indicators in persons with CLD.

## Patient Selection and Characteristics

The sample for this analysis was gathered in early 2013 from individuals who participated in or visited Lyme disease patient-centered online forums in which the survey was posted or publicized. The survey was conducted by LymeDisease.org, a grassroots organization that promotes Lyme disease education and research, and written informed consent was obtained from each subject. Analysis of the survey data was exempted from review by the Carnegie Mellon University Institutional Review Board (IRB) because none of the data contained identifiable personal information. A total of 5,357 subjects responded to the survey, of which a final sample of 3,090 was examined.

**Figure 1:**
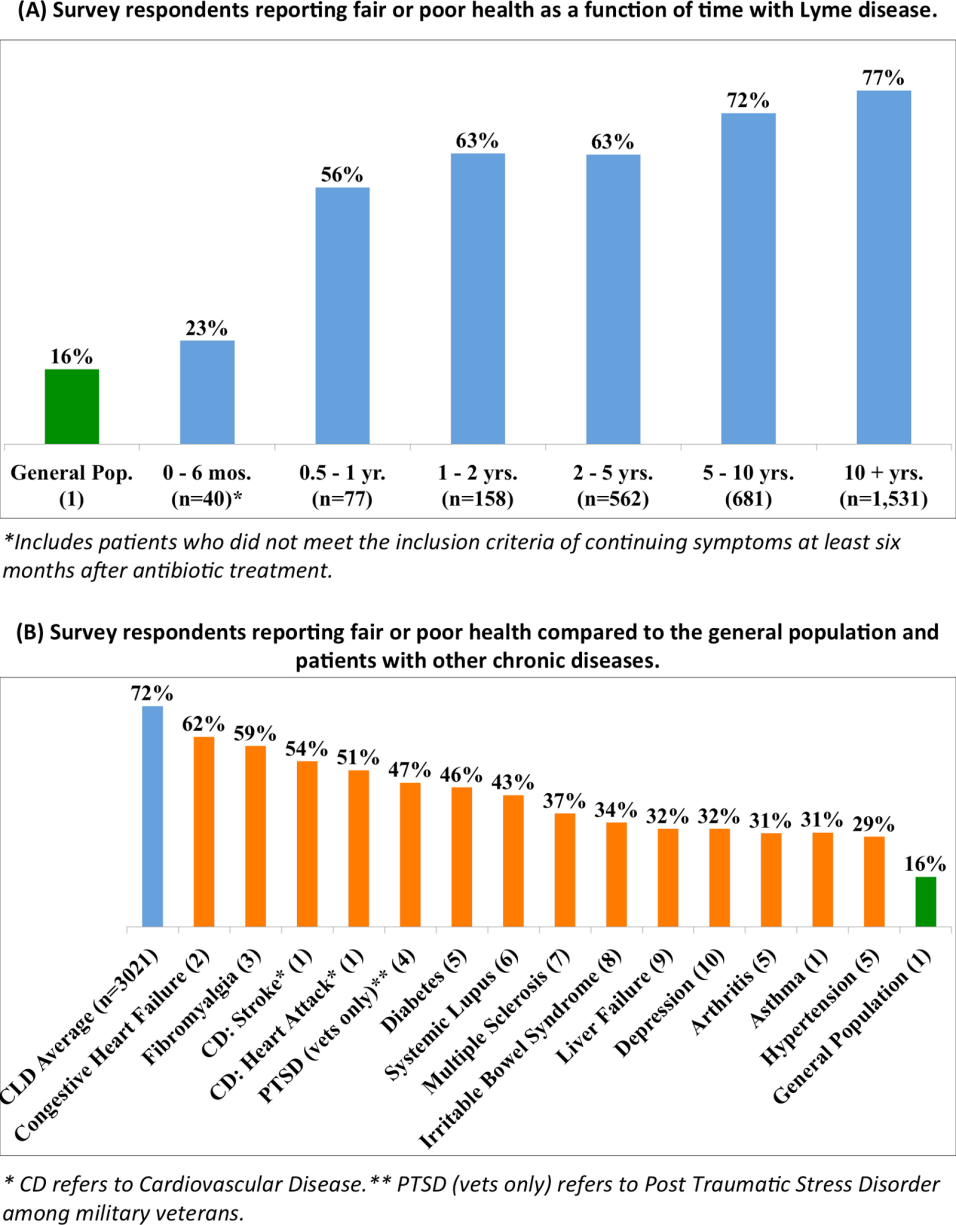
(A) Percentage of survey respondents reporting fair or poor health as a function of length of illness. CLD, chronic Lyme disease. The non-CLD population (0–6 months) is included here to illustrate the progression of disease over time. This population was otherwise excluded from the study (see Table 1). (B) Percentage of survey respondents reporting fair or poor health compared to the general population and patients with other chronic diseases. References: 1. Centers for Disease Control and Prevention (2010d); 2. Burns et al. (1997); 3. Wolfe et al. (1997); 4. Hoge et al. (2007); 5. Centers for Disease Control and Prevention (2009); 6. Yazdany et al. (2011); 7. Becker & Stuifbergen (2009); 8. Lackner et al. (2006); 9. Rangnekar et al. (2013); 10. Fiorillo, Lansky & Bethell (2001).

Table 1 shows the original sample of 5,357 respondents and the exclusion criteria that led to a final sample of 3,090 subjects. To be included in the sample, respondents must have been clinically diagnosed with Lyme disease, have the EM rash and/or have supporting laboratory tests confirming the diagnosis, and have persisting symptoms for more than six months following at least 21 days of antibiotic treatment. An additional cohort of clinically diagnosed Lyme disease patients who had symptoms for less than six months was also identified and included only in the analysis of disease progression over time (see Fig. 1A).

**Table 1:** Study inclusion and exclusion criteria.

Description of sample	Count (% of total sample)
Total sample	5,357 (100%)
Satisfy IRB requirements, not duplicates	5,057 (94.4%)
Clinical diagnosis confirmed by EM rash or positive serology (excluding ELISA or IFA alone)	3,246 (60.6%)
Symptoms persist at least six months after receiving at least 21 days of antibiotic treatment for Lyme disease.	3,090 (57.7%)
Working sample	3,090 (57.7%)

**Notes.**

EM erythema migrans IRB institutional review board ELISA enzyme-linked immunosorbent assay IFA immunofluorescence assay

Respondents whose diagnosis was made clinically without supporting laboratory tests or an EM rash were excluded, and those with only a positive Lyme disease enzyme-linked immunosorbent assay (ELISA) or immunofluorescence assay (IFA) to support their diagnosis were also excluded from the study (a total of 23.8%). A majority of the working sample (a total of 59.5%) was diagnosed by EM rash (6.3%), CDC-positive two-tier test result (ELISA and Western blot, 29.7%), or based on a CDC-positive Western blot alone (23.5%). The rest of the cohort (40.5%) was clinically diagnosed with a positive Western blot using non-CDC interpretive criteria, a positive polymerase chain reaction or culture test, or a positive cerebrospinal fluid test for *B. burgdorferi*. While only 6.3% reported EM rash as the basis of their clinical diagnosis, 39.3% reported having a rash when they contracted the disease.

Table 2 shows the demographic characteristics of the resulting sample. In terms of diagnosis, 7.9% of respondents were not diagnosed until at least 3 months after the onset of symptoms, 16.6% were not diagnosed for at least 6 months, and 61.7% were not diagnosed for at least 2 years. Approximately half (50.5%) of the sample reported having Lyme disease for more than 10 years. Tickborne coinfections confirmed by serological testing were reported by 53.3% of respondents: 23.5% reported at least one co-infection and 29.8% reported two or more co-infections. More specifically, 32.3% of respondents reported laboratory confirmed diagnosis with Babesia, 28.3% with Bartonella, 14.5% with Ehrlichia, 4.8% with Anaplasma, 15.1% with Mycoplasma, 5.6% with Rocky Mountain Spotted Fever, and 0.8% with Tularemia. Recollection of a tick bite was reported by 39.6% of respondents and 39.4% reported a “bulls-eye” or irregular rash.

**Table 2:** Demographic characteristics of respondents.

Variable	Count (% of working sample)
Female	2,364 (82.6%)
Mean age	48.2 years (±12.8)
**Education**	
High school or less	256 (9%)
Some college	751 (26%)
College graduate	1134 (40%)
Graduate school degree	723 (25%)
**Family income**	
Less than $20k	383 (14%)
$20–40k	393 (14%)
$40–60k	440 (16%)
$60–80k	396 (14%)
$80–100k	383 (14%)
$100k +	785 (28%)
**Geography**	
Northeast	984 (35%)
Midwest	371 (13%)
South	673 (24%)
West	813 (29%)

Among respondents, 51.8% were not taking antibiotics. Of those on antibiotics, 92.3% reported taking oral antibiotics, with the remainder taking parenteral antibiotics. Many reasons were given for not being on antibiotics, including using other treatment methods (18.4%), currently well or in remission (10.6%), financial constraints (8.1%), no access to treating physicians (8.0%), treatment no longer helping (7.7%), and treatment side effects (7.4%).

## Study Methods

HRQoL was measured using the CDC 9-item metric (4-item Healthy Days Core Module and 5-item Healthy Days Symptoms Module). Respondents rated their overall health quality as excellent, very good, good, fair or poor. Respondents also answered questions regarding how often during the previous 30 days they experienced vitality, poor physical health, poor mental health, depression, anxiety, and sleep difficulties. In addition, respondents were asked the number of days that their activity was limited due to pain as well as poor physical or mental health. The list of symptoms used in the symptom severity questions were drawn from a review of Lyme research as well as a small online survey that pilot-tested survey questions for a previous study of 2,424 patients regarding access to care (Cairns & Godwin, 2005; Johnson, Aylward & Stricker, 2011; Logigian, Kaplan & Steere, 1990; Shadick et al., 1994; Steere, 1989).

Healthcare utilization was measured using the criteria employed by the Medical Expenditure Panel Survey (Agency for Healthcare Research and Quality, 2014). Visits to doctors or other healthcare professionals, visits to emergency departments, inpatient stays, and home care visits were reported. Additional survey questions were developed regarding severity of symptoms, employment status and clinical presentation of Lyme disease.

## Statistical Analysis

IBM’s SPSS version 21 was used to conduct this analysis. Where scalar variables for HRQoL or healthcare utilization were compared with those of other chronic conditions or the general population, two-tailed *t* tests were performed. Where variables were non-normally distributed, nonparametric Mann–Whitney *U* tests or one-sample Wilcoxon signed rank tests were used. Where subgroups with multiple variables were compared, familywise error rate was addressed by applying Bonferroni or Dunnet T3 corrections, depending on the distribution normality of the variables. Where binary variables were compared, odds ratios with confidence intervals and *p* values were determined. Reported sample sizes and means were used to compute confidence intervals of comparison variables. *P* values <0.05 were considered significant. Correlations between symptoms, time to diagnosis, time since infection, and self-reported health status, all ordinal variables, were analyzed using Spearman coefficients. Ordinal regression analysis was performed to determine the impact of these factors on self-reported health status.

## Results

### CDC HRQoL health module

Table 3 shows the scoring of CLD patients on the HRQoL metric. Respondents reported very poor HRQoL, with a high prevalence of fair or poor self-reported health status. In addition, respondents reported many physically and mentally unhealthy days, many days with activity limitations due to unhealthy days or due to pain, and many days marked by depression, anxiety and lack of rest.

**Table 3:** Nine-item CDC HRQoL metric.

Variable	Mean (S.D.)
**4-item Healthy Days Core Module**	
General health rating (Excellent = 1, Poor = 5)	4.0 (1.0)
Physical health not good (# days out of 30)	20.1 (10.5)
Mental health not good (# days out of 30)	15.5 (10.8)
Physical or Mental health limited usual activities (# days out of 30)	16.8 (11.2)
**5-item Healthy Days Symptoms Module**	
Pain limited activities (# days out of 30)	16.5 (11.7)
Sad, blue or depressed (# days out of 30)	12.4 (10.5)
Worried, tense or anxious (# days out of 30)	15.8 (11.1)
Not enough rest (# days out of 30)	20.3 (10.1)
Very healthy/full of energy (# days out of 30)	3.5 (6.2)

Table 4 compares the HRQoL, health care utilization, and employment impact of those whose diagnosis was based on EM rash or CDC-positive two-tiered serology (EM rash/CDC serology) vs. those whose diagnosis was based on positive CDC Western blot, non-CDC Western blot, PCR, culture and/or spinal tap (other laboratory data). There were no significant differences between the two groups with the exception that patients who reported EM rash/CDC serology were diagnosed more quickly, had a greater number of inpatient stays in the last year, and incurred less out-of-pocket medical expenses. Because there are no significant differences in the majority of metrics and the few differences are slight, the two groups are treated as one for the purpose of this analysis.

**Table 4:** Comparison of patients with diagnosis based on EM rash and/or two tiered serology vs. patients with diagnosis based on other serology.

	Clinical diagnosis based on EM rash and/or two-tiered serology	Clinical diagnosis based on other laboratory data^a^	Full working sample
Count	1087	2003	3090
% of Total	35.2%	64.8%	100.0%
% not diagnosed within 6 months of symptom onset	67.8%^**^	84.1%^**^	78.4%
Fair or Poor General Health	71.6%	72.6%	72.3%
Number of severe or very severe symptoms	3.3	3.2	3.2
At least one severe or very severe symptom	74.3%	71.5%	72.5%
Number of poor physical health days	19.6	20.4	20.1
Number of poor mental health days	15.5	15.5	15.5
Number of visits to a health care professional	19.4	19.4	19.4
Number of visits to the emergency room	1.2	1.0	1.1
% with inpatient stays	18.1%^*^	13.6%^*^	15.2%
% with homecare visits	13.3%	12.5%	12.8%
% with at least $5,000 in out-of-pocket Lyme-related expenses	37.3%^**^	46.4%^**^	43.2%
% who have stopped working	39.4%	42.4%	41.3%
% who have changed work hours or role	28.3%	29.7%	29.2%

**Notes.**

* *P* < 0.01.

** *P* < 0.001.

All other categories had non-significant differences.

a Includes patients diagnosed by CDC positive Western blot, non-CDC positive Western blot, positive PCR, positive culture or positive lumbar puncture.

### Overall health status

Figure 1 shows the comparative health status of patients with CLD. Figure 1A shows that greater time to diagnosis and greater time since infection were significantly correlated with poorer self-reported health status (*ρ* = 0.174 and 0.155, *p* < 0.01 and 0.01, respectively). Only 23% of those with Lyme disease for less than six months reported their healthcare status as poor, compared with 56% at one year and 72% at 5–10 years. Figure 1B compares the health status of survey respondents with other populations. Among CLD patients, 72.3% reported fair or poor health status, significantly exceeding the 16.3% of the general population reporting fair or poor health (*OR* = 13.38, *CI* = 12.35–14.49, *p* < 0.0001). This frequency also significantly exceeds that of other chronic diseases, with congestive heart failure (62%) (Burns et al., 1997) and fibromyalgia (59%) (Wolfe et al., 1997) being the closest in terms of fair or poor health (*OR* = 1.59, *CI* = 1.31–1.94, *p* < 0.0001).

### Symptom severity

**Figure 2:**
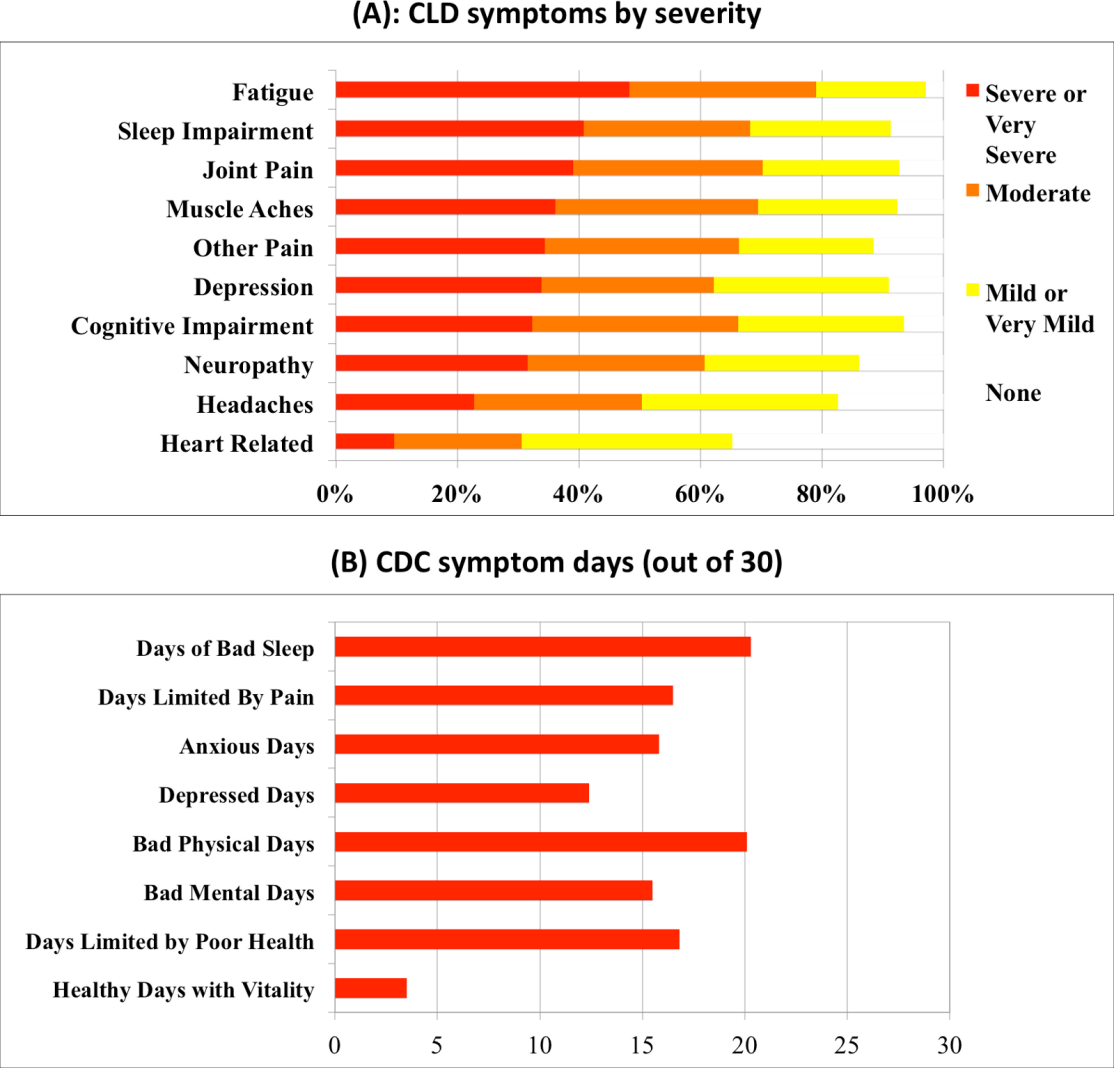
(A) Severity of common respondent symptoms. (B) Frequency of good or bad CDC symptom days per month reported by respondents.

Figure 2 shows the severity of symptoms and number of bad symptom days in patients with CLD. Respondents, on average, reported 3.2 symptoms described as severe or very severe, with 12.7% reporting at least one symptom and 63.3% reporting two or more symptoms as severe or very severe. The 72% of respondents who reported fair or poor health averaged 4.04 severe or very severe symptoms compared with 1.07 among those reporting good, very good, or excellent health.

Figure 2A shows that CLD patients reported the following ten symptoms as severe or very severe: fatigue (48.3%), sleep impairment (40.8%), joint pain (39.1%), muscle aches (36.1%), other pain (34.4%), depression (33.8%), cognitive impairment (32.3%), neuropathy (31.6%), headaches (22.7%) and heart-related issues (9.6%). These symptoms were all significantly correlated with each other, tending to present simultaneously with similar levels of severity (Spearman correlation coefficient range from *ρ* = 0.762 to *ρ* = 0.300, *p* < 0.01). All ten symptoms were significantly correlated with self-reported status (Spearman correlation coefficients from *ρ* = 0.622 to *ρ* = 0.307, *p* < 0.01). Ordinal regression analysis confirmed the ranking of the symptom correlations, identifying fatigue (*ρ* = 0.622, *p* < 0.01), other pain (*ρ* = 0.524, *p* < 0.01), and cognitive impairment (*ρ* = 0.506, *p* < 0.01) as the most impactful vis a vis self-reported health status.

Figure 2B shows that CLD patients also reported a high number of bad symptom days on the CDC symptom module. The number of days with activity limitation due to pain (16.5) reported by CLD patients significantly exceeded days with activity limitation due to pain caused by cancer (13.1), cardiovascular disease (9.2) and emotional problems (8.8) (*t* = 15.73, *p* < 0.001) (Richardson et al., 2008). Bad mental days (15.5) reported by CLD patients were strongly correlated with anxious (15.8) or depressed days (12.4) (*ρ* = 0.737 and 0.791, *p* < 0.01 and 0.01, respectively). The number of unrested days (20.3) reported by CLD patients was associated with the high severity of fatigue reported by these patients (*ρ* = 0.469, *p* < 0.01). The CLD figure significantly exceeded the number of unrested days reported by people with cancer (14.5), cardiovascular disease (11.2) and emotional problems (15) (*t* = 28.87, *p* < 0.001) (Richardson et al., 2008).

### Healthy days and activity limitations

**Figure 3:**
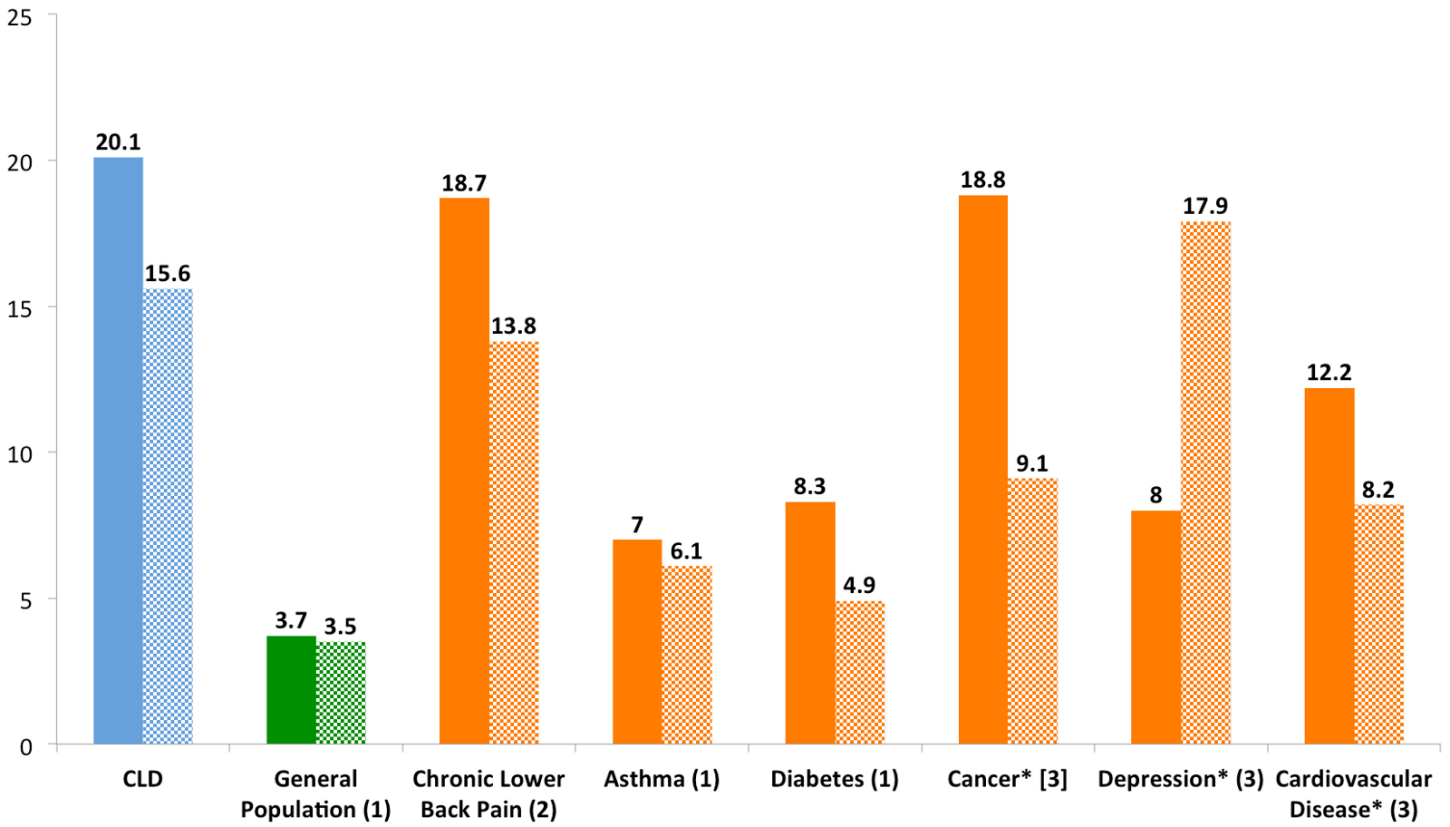
Number of poor physical and mental days per month of patients with CLD compared to the general population and other chronic diseases. References: 1. Centers for Disease Control and Prevention (2010d); 2. Aslan et al. (2010); 3. Richardson et al. (2008).

Figure 3 shows the impact of CLD on physical and mental health. Physical and mental unhealthy days measure how often individuals rated their physical or mental days as not good in the past 30 days. Respondents with chronic Lyme disease were compared to the general population and to patients with other chronic diseases, including patients with chronic conditions severe enough to also have an activity limitation (right side of the chart). Compared to the general population, CLD patients reported significantly more bad physical health days (20.1 vs. 3.7, *t* = 84.6, *p* < 0.001) and significantly more bad mental health days (15.5 vs. 3.5, *t* = 60.3, *p* < 0.001). They also reported more days that poor physical or mental health limited usual self care, recreation or work activities compared to the general population (16.8 vs. 2.3, *t* = 70.2, *p* < 0.001) (graph not shown).

As seen in Fig. 3, the closest chronic disease in terms of both bad physical and mental days was chronic lower back pain (18.7 and 13.8). Cancer patients with an activity limitation had similar bad physical days (18.8), while patients with depression and an activity limitation had similar bad mental days (17.9) (Aslan et al., 2010; Richardson et al., 2008).

### Increased utilization of services

**Figure 4:**
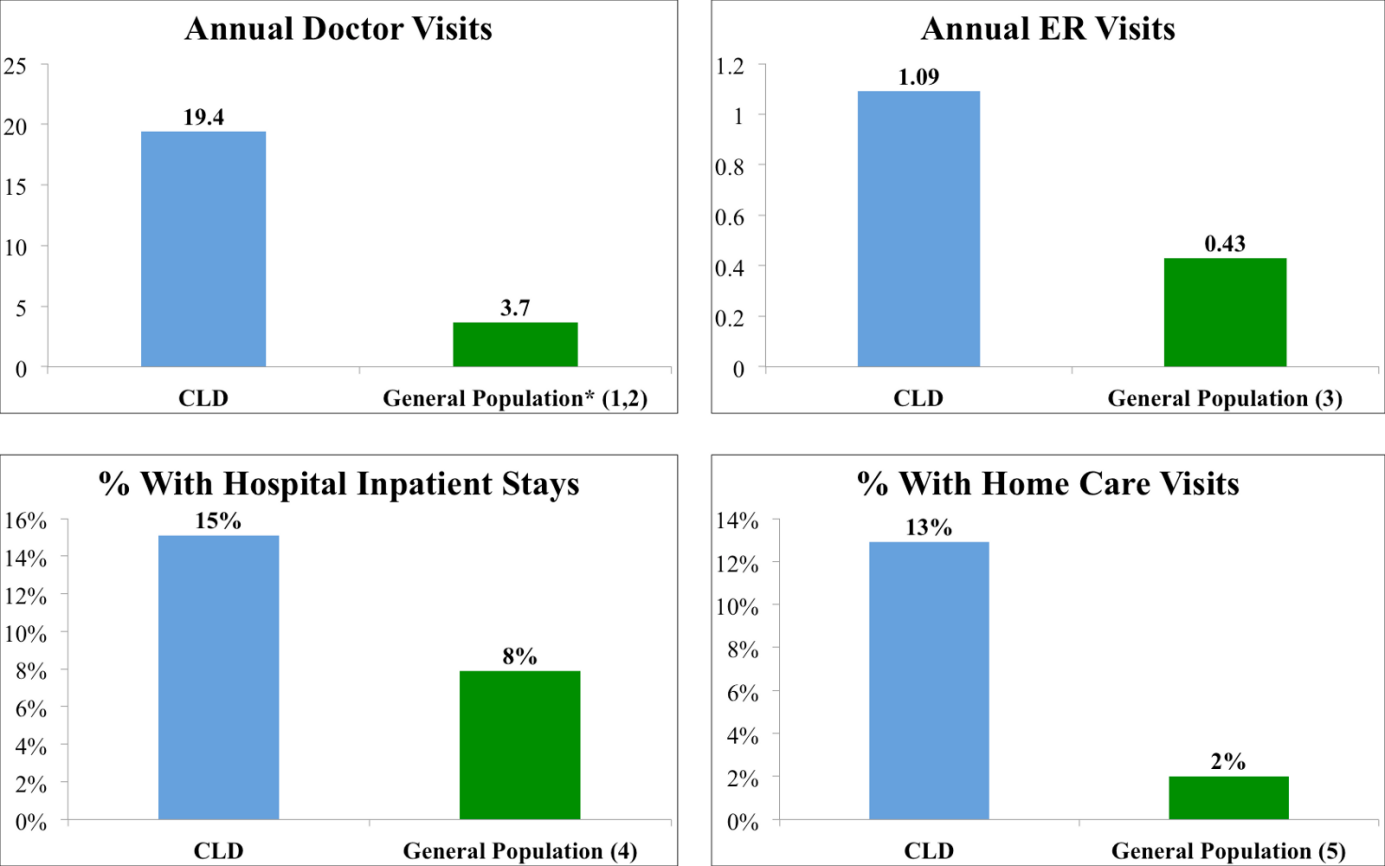
Healthcare services utilization of patients with CLD compared to the general population. References: 1. Centers for Disease Control and Prevention (2010a); 2. Centers for Disease Control and Prevention (2010b); 3. Centers for Disease Control and Prevention (2010c); 4. National Center for Health Statistics (2012b); 5. Agency for Healthcare Research and Quality (2010).

Figure 4 shows that respondents with CLD reported significantly greater healthcare utilization than people in the general population. Compared with the general population, CLD patients visited doctors and healthcare professionals 5 times more often (19.4 vs. 3.7) (Centers for Disease Control and Prevention, 2010a; Centers for Disease Control and Prevention, 2010b) and emergency departments more than twice as often (1.09 vs. .43) (*t* = 32.4 and 10.825, *p* < 0.001 and 0.001, respectively) (Centers for Disease Control and Prevention, 2010c). Furthermore they were almost twice as likely to stay overnight in a hospital (15.2% vs. 7.9%) (National Center for Health Statistics, 2012b) and were roughly six times more likely to receive or pay for homecare visits (12.8% vs. 2.0%) (*OR* = 2.1 and 7.2, *CI* = 1.9–2.3 and 6.4–8.0, *p* < 0.0001 and 0.0001, respectively) (Agency for Healthcare Research and Quality, 2010).

Respondents also reported greater out-of-pocket healthcare-related expenses during the past year compared to the general population. Out-of-pocket expenses included deductibles, copayments, and payments for services not covered by health insurance that were paid by respondents or their families in the past year for costs related to Lyme disease. They did not include over-the-counter remedies. Among CLD respondents, 69% reported spending more than $2,000 and 43% more than $5,000 on out-of-pocket expenses in the preceding year. In comparison, only 20% of the general population exceeded $2,000 annually (including dental costs) and approximately 6% exceeded $5,000 (*OR* = 9.4 and 13.4, *CI* = 8.6–10.2 and 12.3–14.6, *P* < 0.0001 and 0.0001, respectively) (National Center for Health Statistics, 2012a).

### Productivity losses

**Figure 5:**
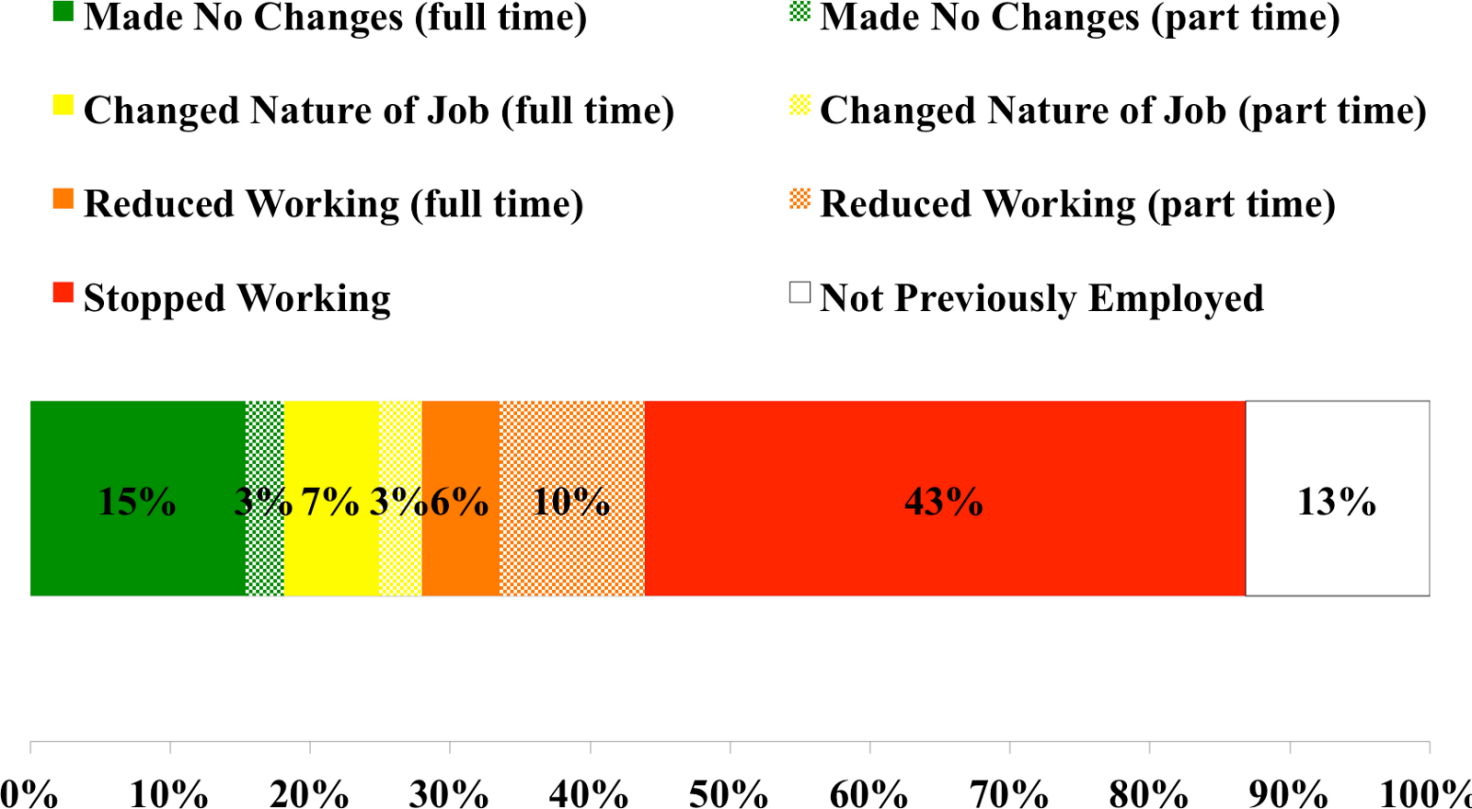
Current employment status of respondents with CLD compared to the general population.

Figure 5 shows the employment status of CLD patients. Respondents suffered greater impairment of their ability to work compared to the general population. In March 2013, 81.0% of the general population ages 25–54 were employed, compared with 45.9% of CLD respondents in that age range (42.1% of the entire adult CLD sample) (*OR* = 0.199, *CI* = 0.181–0.219, *P* < 0.0001) (Toossi, 2012). Approximately 42% of respondents reported that they stopped working as a result of Lyme disease (with 24% reporting that they received disability as a result of CLD), while 25% reported having to reduce their work hours or change the nature of their work due to Lyme disease. These figures compare with 6.3% of the USA population that is unable to work due to health problems and 3.1% that are limited in work due to health problems (*OR* = 10.6 and 10.4, *CI* = 9.8–11.5 and 9.6–11.4, *P* < 0.0001 and 0.0001, respectively) (Centers for Disease Control and Prevention, 2010d). Those respondents who were able to continue working reported missing 15 days of work during the preceding 240-day work year, and they reported an inability to concentrate while at work (so-called presenteeism) during 42 days of work in the preceding year due to illness.

## Discussion

Chronic medical conditions may be characterized by time course, pathogenesis, symptom patterns, late stage manifestations, functional impairment or activity limitation, clinical severity, and management burden on caregivers and society (Institute of Medicine, 2012) These conditions come with varying levels of severity. While some diseases are highly disabling, others are not (Anderson, 2010). This variability makes comparison between diseases problematic. Use of the CDC HRQoL metric provides the opportunity to compare the health status of CLD patients to that of the general population and patients with other chronic diseases despite the variability of these conditions.

Self-rated health is considered to be a more powerful predictor of mortality and morbidity than many objective health measures (New York State Department of Health Disability and Health Program, 2007). How people view their health is strongly correlated with healthcare burden and outcomes. More specifically, people in the general population who regard their health status as fair or poor report more bad physical and mental days, more days with an activity limitation, more bad days due to pain, depression, or anxiety, more sleepless days, higher healthcare utilization and increased medical expenses (Moriarty, Zack & Kobau, 2003). They also experience fewer days when they are full of energy/vitality (Moriarty, Zack & Kobau, 2003).

As noted in Fig. 1, patients with CLD reported fair or poor health status significantly more frequently (72%) compared to people in the general population (16%) and patients with other chronic conditions. Consistent with this compromised health status, CLD patients reported a significant symptom disease burden (Fig. 2), more bad physical and mental health days compared to the general population and most other chronic diseases reviewed here (Fig. 3), and increased utilization of healthcare services resulting in more medical costs compared to the general population (Fig. 4). CLD patients also reported greater activity limitations and impairment in their ability to work (Fig. 5). The degree of compromised health reported by respondents is also reflected by the need for special medical aids. Approximately 22.8% of respondents reported requiring special medical equipment as a result of Lyme disease, with 17.2% requiring the use of either a cane (13.7%) and/or a wheelchair (6.8%).

Approximately 75% of survey respondents reported severe or very severe symptoms related to fatigue, joint pain, headaches, other pain, muscle aches, neuropathy, cognitive impairment, sleep impairment or mood impairment, and 63% reported more than one symptom as severe or very severe (Fig. 2). The strongest driver of health status in our sample was fatigue. Chandra and colleagues noted moderate to severe levels of fatigue and pain in their sample of patients with CLD, and they also found that fatigue was the primary driver of poor physical component scores in their patients (Chandra, Keilp & Fallon, 2013). In a recent study by Aucott et al. (2013), 20–45% of Lyme disease patients who were diagnosed and treated early reported fatigue, widespread pain or sleep disturbance six months later. Moreover, in accordance with the Aucott study, the frequency of fatigue and sleep impairment reported here exceeded that found in the general population, while the frequency of pain exceeded the level reported in fibromyalgia (Aucott et al., 2013). The severity of symptoms reported here also mirrors the findings of Klempner et al. (2001) that patients with CLD may suffer a degree of disability equivalent to that of patients with congestive heart failure.

Compared to the general population and patients with other chronic diseases, CLD respondents reported significantly more bad physical and mental health days (Fig. 3). They also reported more days in which poor physical or mental health limited usual self care, recreation or work activities compared to the general population. CLD respondents reported more bad physical days than patients with chronic low back pain, asthma, diabetes, cancer, depression and cardiovascular disease, and more bad mental days than patients with each of these diseases with the exception of depression. In a study using a different quality of life scale, the mental and physical component scores of patients with CLD were found to be worse than the scores of patients with heart disease, diabetes, depression, cancer and osteoarthritis (Cameron, 2008). Moreover, like other chronic physical diseases, the number of bad physical days was significantly higher than the number of bad mental days (Cameron, 2008; Fiorillo, Lansky & Bethell, 2001).

Chronic illnesses account for 84% of healthcare costs, and those with chronic illnesses are the greatest users of healthcare services (Anderson, 2010). One study found that people in the highest 5 percent of the medical expense category were 11 times more likely to be in fair or poor physical health (Stanton, 2006). Furthermore, the costs for patients with an activity limitation are roughly double those of patients without an activity limitation (Anderson, 2010). Compared with the general population, CLD patients were five times more likely to visit doctors and healthcare professionals and more than twice as likely to be seen in an emergency department. In addition, they were almost twice as likely to stay overnight in a hospital and roughly six times more likely to receive or pay for home care visits (Fig. 4). This markedly increased healthcare utilization is undoubtedly associated with increased healthcare costs, although this study was not designed to measure total medical expenditures.

We also found that patients with CLD incurred high out-of-pocket expenses compared with other diseases. The percentage of CLD patients spending in excess of $5,000 in out-of-pocket costs was 46% compared to 5% in the general population. This may reflect the fact that CLD patients had greater co-payments as a result of more frequent physician visits. In addition, it is likely that these physician visits would be out of network because many insurers have adopted restrictive Lyme disease guidelines and may not cover extended therapy with antibiotics or other medications often used for CLD treatment.

As noted in Table 4, respondents who reported that their diagnosis was based on EM rash/CDC serology were diagnosed more often within six months (84.1% vs. 67.8%) but incurred more inpatient stays (18.1% vs. 13.6%) than patients diagnosed by other laboratory data. In addition, respondents who were diagnosed by other laboratory data had a higher rate of annual out-of-pocket costs exceeding $5,000 (46.4% vs. 37.3%). The reason for this difference is unclear and requires further study. A possible explanation for the fact that those diagnosed by other laboratory data had fewer inpatient stays may be that they avoided such stays because they were bearing a greater portion of costs due to insurance denials.

While good health affects individual well-being and healthcare utilization, it also affects employee productivity. Approximately 80% of the cost of chronic illness is productivity losses (Devol & Bedroussian, 2007). As might be expected, the ability of CLD patients to retain full time work was adversely affected as a result of their large number of days with activity limitations due to poor health (Fig. 5). A substantial percentage of CLD patients reported that their Lyme disease impaired their ability to work, resulting in either a reduction in work hours, a modification of the type of work performed or quitting work altogether. Other studies have found that a quarter of CLD patients were on disability due to their disease at some point in their illness, with most disability lasting more than two years (Johnson, Aylward & Stricker, 2011). Respondents reported an average of two healthcare-related visits per month, which also adversely impacted their ability to work.

While it is beyond the scope of this study to calculate the economic cost of this loss of productivity, it would appear to be substantial. Loss of productivity results when chronically ill workers are unable to work, reduce their work hours, take excessive sick days or perform below par while at work. Diminished work performance exacts a toll on the worker, the worker’s family and the employer. Ultimately, the government and society also suffer because of the reduction in productivity of these compromised workers (Devol & Bedroussian, 2007).

Internet surveys can be effectively delivered to the general population on a large scale. An increasing number of survey studies have turned to the internet because of the high costs and decreased response rates for telephone surveys, mail surveys, and face-to-face interviews, and online methods of data collection have been found to be comparable to traditional methods (Liu et al., 2010; van Gelder, Bretveld & Roeleveld, 2010). Moreover, unlike more objective medical outcomes, HRQoL survey data must be collected directly from the patient, which makes internet data collection ideal (Bhinder et al., 2010).

An important consideration in internet surveys is selection bias, which may impact generalizability (Liu et al., 2010). Researchers control for this bias by using demographically balanced panels and by using weighting adjustments to compensate for response variation from known population values (Liu et al., 2010). In Lyme disease, however, these methods are not practical for two reasons. First, the normal population distribution and clinical characteristics of patients and CLD are not well defined, and the generalizability of research findings to the clinical population has been problematic. Second, the relatively low rate of Lyme disease reporting under the CDC surveillance system would require extraordinarily large demographically balanced panels to achieve a meaningful sample size.

As the CDC’s recent revision of the annual incidence of Lyme disease from 30,000 to more than 300,000 cases suggests, the true incidence and spectrum of disease is still in an emerging state. The CDC’s revision of the surveillance case numbers arose from their preliminary review of three CDC funded studies that did not utilize the restrictive CDC surveillance criteria, but rather relied on self-reported diagnosis, insurance coding by healthcare providers, and positive serology. Hence, the increased incidence numbers suggest that the surveillance system does not reflect the full spectrum of the disease seen clinically.

The 30,000 patients captured annually in the surveillance system are outnumbered ten-to-one by those who are not, and the difference between those counted as cases and the 270,000+ who are not is unknown. Whether the population demographics that emerge upon further study conform to those currently represented in the CDC surveillance system remains to be seen. Points of variation may include clinical characteristics (e.g., presence of EM rash or symptom profiles) or demographic characteristics (e.g., age, gender, or geographic distribution of reported cases). These issues negatively impact the ability and desirability of weighting the sample to reflect a normal distribution of the patient population.

In this study 39.3% of patients reported the presence of an EM rash compared to 69% of cases included in the CDC surveillance numbers (Centers for Disease Control and Prevention, 2008). Possible reasons for this discrepancy include (a) patients may not recall an EM rash that was present, (b) the CDC surveillance reporting system may have an implicit selection bias toward those with an EM rash, or (c) patients without an EM rash may be less likely to receive treatment that would prevent progression of the disease. For example, Aucott reports that 54% of Lyme disease patients who present without a rash are misdiagnosed (Aucott et al., 2009). Physicians may fail to diagnose Lyme disease when the EM rash is irregular or homogeneous rather than the textbook “bulls-eye” shape (Schutzer et al., 2013; Smith et al., 2002). It is also important to recognize that even among the CDC surveillance cases there is considerable variation in the reported incidence of EM rash in different parts of the USA, ranging from 51% to 87% depending on the state (Centers for Disease Control and Prevention, 2008). In addition, incidence by age and sex distribution of CDC surveillance cases vary by state (Centers for Disease Control and Prevention, 2008). It is not known whether these clinical and demographic variations reflect reporting anomalies or geographical strain diversity of *B. burgdorferi*.

Generalizability issues have complicated the four National Institutes of Health (NIH)-funded randomized controlled trials on CLD, where researchers screened large numbers of patients to yield small sample sizes (Bhinder et al., 2010). Indeed, one study screened 3,368 patients to yield 36 patients who met the restrictive entry criteria (Fallon et al., 2008). The trade-off between internal validity and external generalizability in clinical trials is widely recognized (Bhinder et al., 2010; Donkin et al., 2012). However while 25% of those with other diseases may meet the entry criteria for a clinical trial, only 10% of those with CLD meet these restrictive entry criteria (Donkin et al., 2012).

Our findings suggest that the CDC and NIH should include questions regarding Lyme disease in national population surveys that would permit researchers to accurately characterize the annual incidence, prevalence, and demographic distribution of Lyme disease in the United States. However, in the meantime, it is important to recognize that generalizability problems are common given the current state of the science in Lyme disease. While our study does not seek to characterize a well-defined population of patients, it provides important insight into the clinical characteristics of the emerging CLD population.

## Strengths and Limitations

This study has several strengths. First, to our knowledge it is the first study to evaluate CDC HRQoL of patients with CLD. Second, the sample size of the study was robust and permitted analysis of multiple patient variables. Third, the use of a standardized questionnaire allowed in-depth analysis of CLD patients as well as measurable comparison between these patients, the general population and other chronic diseases. Finally, despite the exclusion criteria utilized in the study, the sample population was much more diverse than the populations examined in published randomized controlled trials of CLD. For example, our sample included patients with tickborne coinfections who were generally not evaluated in other Lyme disease studies. Thus the study results should be more generalizable to the broad spectrum of CLD patients compared to the results of the limited randomized controlled trials performed to date (Bhinder et al., 2010; Liu et al., 2010).

There are limitations to our study. First, our sample is self-selected from participants who are sick enough (and Internet-savvy enough) to seek online support for their illness. Because respondents were not a randomly drawn sample, the results may not be fully representative of persons living with Lyme disease in the United States. Second, respondents reported a large number of activity limitation days. It has been noted that patients limited by a condition may represent the most severely ill individuals with that condition (Richardson et al., 2008). This matches our expectation that our sample represents people who are more sick rather than less sick. Along these lines, survey results were limited to patients with CLD who reported persistent symptoms for six months or more. Patients with acute Lyme disease who are diagnosed and treated early would be expected to have less quality of life impairment, as noted in Fig. 1A.

A third limitation is that the results are based on self-reported information without diagnostic confirmation. However, self-reported information has been found to have acceptable levels of reliability when compared to medical chart information (Bayliss et al., 2012). Moreover, self-rated health is considered to be a reliable indicator of perceived health and personal well-being and may be a more powerful predictor of mortality and morbidity than many objective measures of health (New York State Department of Health Disability and Health Program, 2007).

A fourth limitation is that the sample population is slightly older (about 1.5 years) than the general population, and this difference may impact both health status and utilization of health services. In addition, the existence of comorbidities, which may increase reported health status and healthcare utilization was not addressed in the survey. The high percentage of women in this sample may reflect the higher percentage of females seen in some CLD studies as well as the female-skewed demographics often seen in patient survey responses (Boscardin & Gonzales, 2013; Stricker & Johnson, 2009).

## Conclusions

Based on our survey results, CLD patients suffer from significantly impaired HRQoL, utilize healthcare services more frequently and have greater limitations on their ability to work compared to the general population and patients with other chronic diseases. The heavy burden of illness associated with CLD highlights the need for earlier diagnosis of Lyme disease to avoid progression to CLD, as well as the need for innovative treatment approaches to reduce the burden of illness and concomitant costs posed by this illness.

## Supplemental Information

10.7717/peerj.322/supp-1 Supplemental Information 1 Dataset
